# Treatment of Human Glioblastoma with a Live Attenuated Zika Virus Vaccine Candidate

**DOI:** 10.1128/mBio.01683-18

**Published:** 2018-09-18

**Authors:** Qi Chen, Jin Wu, Qing Ye, Feng Ma, Qian Zhu, Yan Wu, Chao Shan, Xuping Xie, Dapei Li, Xiaoyan Zhan, Chunfeng Li, Xiao-Feng Li, Xiaoling Qin, Tongyang Zhao, Haitao Wu, Pei-Yong Shi, Jianghong Man, Cheng-Feng Qin

**Affiliations:** a State Key Laboratory of Pathogen and Biosecurity, Institute of Microbiology and Epidemiology, Academy of Military Medical Sciences, Beijing, China; b State Key Laboratory of Proteomics, National Center of Biomedical Analysis, Beijing, China; c Guangzhou No.8 People's Hospital, Guangzhou Medical University, Guangzhou, China; d Center for Systems Medicine, Institute of Basic Medical Sciences, Chinese Academy of Medical Sciences & Peking Union Medical College, Beijing, China; e Suzhou Institute of Systems Medicine, Suzhou, China; f Department of Neurobiology, Beijing Institute of Basic Medical Sciences, Beijing, China; g Department of Biochemistry and Molecular Biology, Department of Pharmacology and Toxicology, Sealy Center for Structural Biology & Molecular Biophysics, University of Texas Medical Branch, Galveston, Texas, USA; h Tianjin Key Laboratory of Risk Assessment and Control Technology for Environment and Food Safety, Institute of Environmental Medicine, Academy of Military Medical Sciences, Tianjin, China; Mailman School of Public Health, Columbia University

**Keywords:** Zika virus, anticancer therapy, glioblastoma, vaccine

## Abstract

Glioblastoma (GBM) is the deadliest type of brain tumor, and glioma stem cells (GSCs) contribute to tumor recurrence and therapeutic resistance. Thus, an oncolytic virus targeting GSCs may be useful for improving GBM treatment. Because Zika virus (ZIKV) has an oncolytic tropism for infecting GSCs, we investigated the safety and efficacy of a live attenuated ZIKV vaccine candidate (ZIKV-LAV) for the treatment of human GBM in a GSC-derived orthotopic model. Intracerebral injection of ZIKV-LAV into mice caused no neurological symptoms or behavioral abnormalities. The neurovirulence of ZIKV-LAV was more attenuated than that of the licensed Japanese encephalitis virus LAV 14-14-2, underlining the superior safety of ZIKV-LAV for potential GBM treatment. Importantly, ZIKV-LAV significantly reduced intracerebral tumor growth and prolonged animal survival by selectively killing GSCs within the tumor. Mechanistically, ZIKV infection elicited antiviral immunity, inflammation, and GSC apoptosis. Together, these results further support the clinical development of ZIKV-LAV for GBM therapy.

## Abstract (precis)

Glioblastoma (GBM), the deadliest type of brain tumor, is currently incurable because of its high recurrence rate after traditional treatments, including surgery to remove the main part of the tumor and radiation and chemotherapy to target residual tumor cells. These treatments fail mainly due to the presence of a cell subpopulation called glioma stem cells (GSCs), which are resistant to radiation and chemotherapy and capable of self-renewal and tumorigenicity. Because Zika virus (ZIKV) has an oncolytic tropism for infecting GSCs, we tested a live attenuated ZIKV vaccine candidate (ZIKV-LAV) for the treatment of human GBM in a human GSC-derived orthotopic model. Our results showed that ZIKV-LAV retained good efficacy against glioblastoma by selectively killing GSCs within the tumor. In addition, ZIKV-LAV exhibited an excellent safety profile upon intracerebral injection into the treated animals. The good balance between the safety of ZIKV-LAV and its efficacy against human GSCs suggests that it is a potential candidate for combination with the current treatment regimen for GBM therapy.

## INTRODUCTION

Zika virus (ZIKV) is an enveloped, single-stranded, positive-sense RNA virus of the genus *Flavivirus* within the family *Flaviviridae*. Like ZIKV, many flaviviruses, including dengue virus (DENV), yellow fever virus (YFV), West Nile virus (WNV), Japanese encephalitis virus (JEV), and tick-borne encephalitis virus (TBEV), are significant human pathogens. The recent epidemics of ZIKV in the Americas have caused a global public health emergency because of its unexpected causal link to microcephaly and other congenital diseases in fetuses from infected pregnant women (1–4). ZIKV preferentially infects neural progenitor cells (NPCs), causing cell death and reduced proliferation, which results in impaired brain development in the fetus (5, 6). In response to the public health emergency, the scientific community has rapidly developed a number of promising ZIKV vaccines with good safety and efficacy profiles in mice and nonhuman primates, and some of the vaccines have already entered phase I/II clinical trials (7, 8).

Glioblastoma (GBM) is the most common and malignant form of primary brain tumor, and despite aggressive treatment with surgery, radiation, and chemotherapy, GBM remains lethal, with a median survival time of less than 2 years (9, 10). Thus, novel treatment options are urgently needed. Glioma stem cells (GSCs) have self-renewal, tumorigenic and differentiation potential, and are thus similar to NPCs in that respect. GSCs play critical roles in GBM progression, recurrence, and therapeutic resistance and have been well recognized as a major therapeutic target for GBM (11–13). Oncolytic viruses are emerging as promising therapeutic options, and multiple versions of oncolytic viruses have been explored for virotherapy against human GBM, with promising results (14). Treatment of grade IV malignant glioma patients with a recombinant polyrhinovirus chimera was recently shown to improve their survival rates (15).

The specific tropism of ZIKV for NPCs in the fetus has raised the possibility of its use as an oncolytic virus to target GSCs. Zhu et al. recently demonstrated that ZIKV was highly efficient at infecting and killing human GSCs *in vitro*. Treatment with a mouse-adapted ZIKV Dakar strain led to a regression of mouse glioma and prolonged the survival of mice engrafted with glioblastoma cells (16). However, whether ZIKV is efficacious for treating patient-derived GBM remains to be determined. Such *in vivo* efficacy is required before ZIKV can progress into the clinic as a virotherapy against human GBM.

With respect to its potential use as a therapeutic agent, the pathological diseases associated with intracerebral injection of ZIKV into the brain have raised safety concerns. One approach to addressing this safety barrier is to engineer ZIKV in a manner that eliminates its virulence but maintains its oncolytic activity against GBM. Along these lines, we have recently developed a genetically modified live attenuated ZIKV vaccine (ZIKV-LAV) that contains a 10-nucleotide deletion in the 3′ untranslated region (3′UTR) of the viral genome (17). The genetic stability and safety profile of ZIKV-LAV have been well characterized in mice and nonhuman primates (18). The goals of this study were to (i) profile the safety of ZIKV-LAV for intracerebral injection, (ii) evaluate the *in vivo* efficacy of ZIKV-LAV against GBM in a patient-derived GSC orthotopic mouse model, and (iii) define the oncolytic mechanism of ZIKV-LAV during GBM treatment.

## RESULTS

### Intracerebral administration of ZIKV-LAV is safe in multiple mouse models.

**FIG 1:**
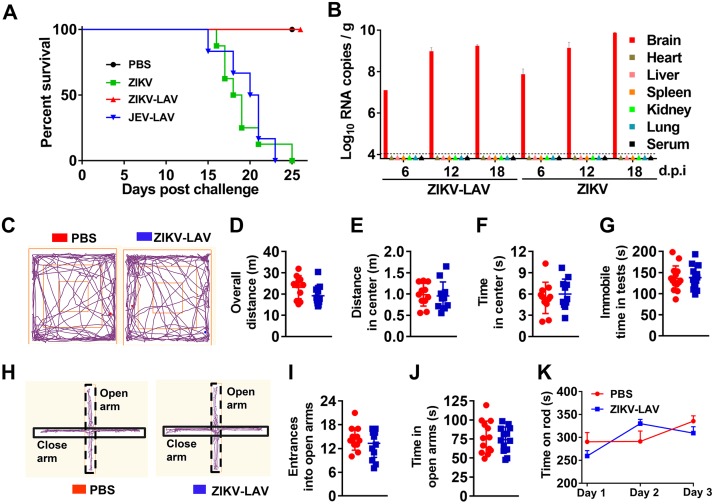
Characterization of the safety profile of ZIKV-LAV upon intracerebral administration in mice. (A) Groups of 3-week-old BALB/c nude mice (*n* = 30) were intracerebrally inoculated with 10,000 PFU of ZIKV-LAV, ZIKV, JEV-LAV, or PBS. The animals were monitored daily for clinical signs and survival. (B) Tissue distribution of ZIKV-LAV and wild-type ZIKV in nude mice following intracerebral administration. Sera and tissues were collected at the indicated days postinfection (d.p.i) (*n* = 3 per time point), and viral RNA copies were detected using RT-qPCR. The triangle symbols denote no detectable viral RNA. (C to K) Behavior evaluations of mice receiving intracerebral administration of ZIKV-LAV. Two groups of 4-week-old C57 mice were intracerebrally inoculated with ZIKV-LAV or PBS. The behavior tests were performed 14 days after injection. (C to F) Open field test (*n* = 11). (C) Mouse traces were recorded in the open field by an infrared video tracking system for 5 min. (D) Overall distances the mice traveled in the open field. (E) Distances the mice traveled in the center of the open field. (F) Total times the mice traveled in the center of the open field. (G) Tail suspension test (*n* = 14). The total times that the PBS-inoculated and ZIKV-LAV-infected mice were immobile during the tail suspension test during a 6-min time period are presented. (H to J) Elevated plus maze test (*n* = 13). (H) Mouse traces in the elevated plus maze test were recorded by an infrared video tracking system for 5 min. (I) Entrances of mice into open arms. (J) Total times that the mice traveled in the open arms. Statistical analysis was performed using unpaired *t* tests. For all the analyzed parameters, the differences between the ZIKV-LAV-infected and PBS control groups were statistically nonsignificant, with a *P* value of >0.05. (K) Rota-rod test (*n* = 14). All animals were subjected to one session per day for 3 days. The total times that the PBS (mock)-treated and ZIKV-LAV-infected mice spent on the rod were compared by two-way ANOVA, with a *P* value of >0.05.

We previously showed that ZIKV-LAV was highly attenuated in newborn CD-1 mice upon intracerebral injection even at a dose of 10,000 PFU; no morbidity or mortality was detected in any of the infected mice (17). To further examine the neurovirulence safety, we intracranially injected 10,000 PFU of ZIKV-LAV (derived from preepidemic Cambodian strain FSS 13025) and wild-type ZIKV (the epidemic GZ01 strain isolated in China from an imported human patient in 2016) into 3-week-old BALB/c nude mice. As a positive control, we also infected the mice with the same dose of a clinically licensed JEV-LAV that has been used in more than 400 million children under the age of 15 years in China (19). As expected, all the nude mice succumbed to the wild-type ZIKV infection within 25 days, exhibiting typical neurological symptoms and body weight loss; even administration of the licensed JEV-LAV resulted in neurovirulence in the nude mice (Fig. 1A; see also Fig. S1A in the supplemental material). In contrast, ZIKV-LAV did not cause any morbidity, mortality, or weight loss in the infected animals (Fig. 1A; see also Fig. S1A). Interestingly, both wild-type ZIKV and ZIKV-LAV showed tropism for the brain, and viral RNA accumulation was observed in the brains of the infected mice until 18 days postinjection, whereas no viral RNAs were detected in the serum or in other tested peripheral organs, including the heart, lung, liver, spleen, and kidney (Fig. 1B). In addition, no pathological changes were observed in the brains of ZIKV-LAV-injected mice by histopathological examination (Fig. S1B). However, the pathological outcomes may have been affected by limited inflammation in the nude mice due to the lack of T cells. Sequencing the viruses recovered from the mouse brains on day 24 postinjection confirmed the retention of the 10-nucleotide deletion in the 3′UTR of the viral genome (Fig. S1C).

10.1128/mBio.01683-18.1 FIG S1 Characterization of the safety profile of ZIKV-LAV upon intracranial administration in mice. (A) Groups of 3-week-old BALB/c nude mice (*n* = 30) were intracranially inoculated with 10,000 PFU of ZIKV-LAV, ZIKV, JEV-LAV, or PBS. The animals were monitored daily for weight change. (B) Histological examination of the brain tissues of nude mice intracerebrally inoculated with 10,000 PFU of ZIKV-LAV on day 7 postinoculation. PBS injection was used as a mock control. At least three brain sections were prepared for each mouse, and two or three animals were dissected from each group. (C) Viral RNAs isolated from the brain tissues of BALB/c nude mice inoculated with ZIKV-LAV were sequenced for detection of the 10-nucleotide deletion from the 3′UTR of the viral genome. Download FIG S1, TIF file, 2.9 MB. Copyright © 2018 Chen et al. 2018 Chen et al. This is an open-access article distributed under the terms of the Creative Commons Attribution 4.0 International license.

A recent study demonstrated that ZIKV infection could cause behavioral abnormalities in multiple animal models (20). To further evaluate the neurovirulence of ZIKV-LAV, we performed four sets of behavioral tests to examine the potential adverse effects on ZIKV-LAV-infected mice, including the standard open field test (Fig. 1C to F), tail suspension test (Fig. 1G), elevated plus maze test (Fig. 1H to J), and rota-rod test (Fig. 1K). Four-week-old C57 mice were intracranially injected with 10,000 PFU of ZIKV-LAV, and all the infected animals remained healthy without any symptoms or behavioral abnormalities (e.g., diarrhea, inappetence, depression, inactivity, and self-injury) during the observation period. The ZIKV-LAV-infected animals showed anxiety levels and motor functions similar to those of animals in the mock control group (*P  > *0.05) during the four behavior tests (Fig. 1C to K). Collectively, these results showed that the intracerebral administration of ZIKV-LAV to mice did not cause detectable behavioral abnormalities, neurovirulence, or virus spillover to other organs, suggesting an excellent safety profile for ZIKV-LAV during oncolytic virotherapy.

### ZIKV-LAV impairs GBM formation and prolongs animal survival.

**FIG 2:**
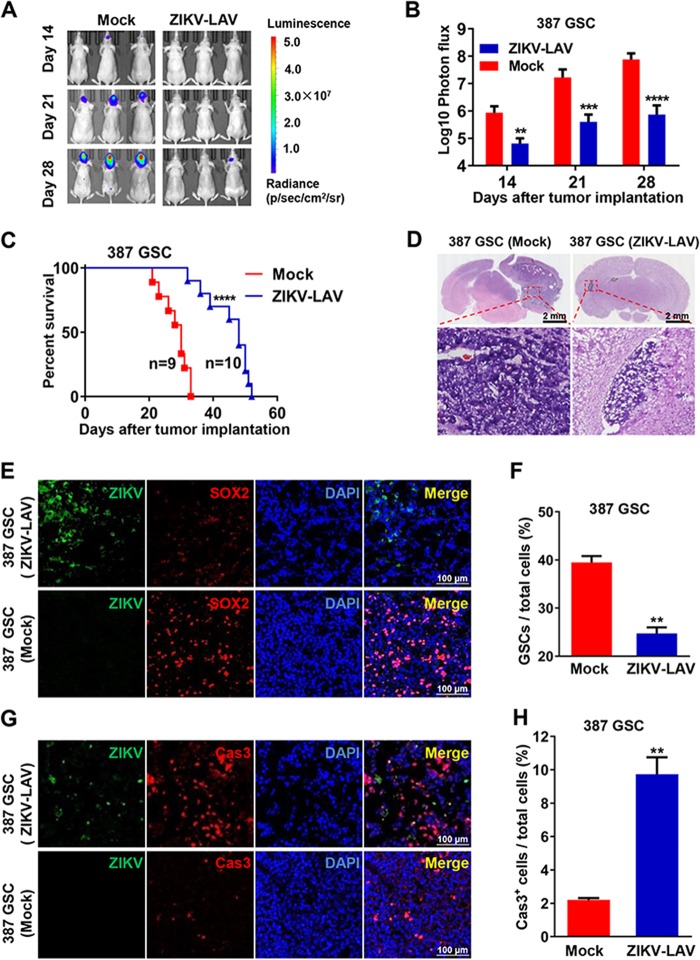
ZIKV-LAV inhibited GSC tumor growth and prolonged animal survival. (A to C) Three-week-old BALB/c nude mice were intracerebrally transplanted with 50,000 luciferase-labeled type 387 GSCs mixed with 10,000 PFU of ZIKV-LAV or RPMI 1640 (Mock). (A) *In vivo* bioluminescence imaging of tumor growth from luciferase-labeled 387 GSCs at the indicated times. (B) Quantification of photon flux from ROI analysis of the dorsal side. Statistical significance was analyzed by unpaired Student’s *t* tests. **, *P* < 0.01; ***, *P* < 0.001; ****, *P* < 0.0001. (C) Survival curves. Statistical significance was determined by the log rank test. ****, *P* < 0.0001. (D to H) Three-week-old BALB/c nude mice transplanted with 387 GSCs mixed with ZIKV-LAV or RPMI 1640 (Mock) were sacrificed on day 23 postimplantation. The brain tissues were collected and prepared for cryosectioning. (D) Representative images of brain sections stained with hematoxylin and eosin. The tumor areas are enclosed with black lines. (E) Immunofluorescence staining of cryosections for the ZIKV E protein (green), SOX2 (red), and DAPI (blue). The percentages of GSCs in tumor tissues are shown as means ± SD (*n* = 3; right panel). **, *P* < 0.01. (F) Cleaved caspase 3 (Cas3) staining of apoptotic cells in tumor tissues. The percentages of Cas3^+^ cells in tumor tissues are shown as means ± SD (*n* = 3; right panel). **, *P* < 0.01.

The excellent safety profile of ZIKV-LAV prompted us to test its therapeutic efficacy against human GBM in a GSC-derived orthotopic mouse model (21–23). These GSCs have been functionally validated in previous studies and shown to form orthotopic xenografts in mice that closely resemble the genotype and phenotype of human GBM (21, 24, 25). Two GSC lines (387 GSCs and 4121 GSCs) that stably express firefly luciferase were co-implanted with ZIKV-LAV (26); we chose to transplant a mixture of GSCs (50,000 cells) and ZIKV-LAV (10,000 PFU) to mimic clinical treatment. After removal of the main tumor by surgery, residual GSCs may remain in proximity to the original resection cavity. Bioluminescence imaging showed that brain tumors progressed rapidly in animals implanted with 387 GSCs (Fig. 2A and B) and 4121 GSCs (Fig. S2A and B) following implantation, and all animals exhibited neurological symptoms and died (Fig. 2C). In contrast, co-implantation with ZIKV-LAV significantly delayed tumor growth and progression (Fig. 2A and B; see also Fig. S2A and B). In addition, the co-implantation with ZIKV-LAV also increased the median survival duration from 30 days to 48 days for the 387 GSC-implanted mice (Fig. 2C) and from 31 days to 53 days for the 4121 GSC-implanted animals (Fig. S2C).

10.1128/mBio.01683-18.2 FIG S2 ZIKV-LAV inhibited GSC tumor growth and prolonged animal survival by infecting and killing GSCs. (A to C) Three-week-old BALB/c nude mice were transplanted with 50,000 luciferase-labeled type 4121 GSCs mixed with 10,000 PFU ZIKV-LAV or RPMI 1640 (mock) by the intracranial route. (A) *In vivo* bioluminescence imaging of gliomas from luciferase-labeled 4121 GSCs at the indicated times. (B) Quantification of the photon flux from ROI analysis of the dorsal side. Statistical significance was analyzed by Student’s *t* tests. ****, *P* < 0.0001. (C) Survival curves. Statistical significance was determined by the log rank test using GraphPad Prism 7.01. ****, *P* < 0.0001. (D to H) Three-week-old BALB/c nude mice transplanted with 4121 GSCs mixed with ZIKV-LAV or RPMI 1640 (mock) were sacrificed on day 25 postimplantation. The brain tissues were collected for cryosectioning and analysis. (D) Representative images of brain tissue sections stained with hematoxylin and eosin. The tumor areas are enclosed with black lines. Download FIG S2, TIF file, 2.9 MB. Copyright © 2018 Chen et al. 2018 Chen et al. This is an open-access article distributed under the terms of the Creative Commons Attribution 4.0 International license.

Histological examination of brain sections by hematoxylin and eosin staining confirmed that all the tested GSC-implanted animals (*n* = 8) had developed typical invasive gliomas on day 23 postimplantation with 387 GSCs and on day 25 postimplantation with 4121 GSCs. In contrast, visible tumors were observed in only a small proportion (2 of 8) of ZIKV-LAV-treated animals, and the tumor sizes were significantly reduced (Fig. 2D; see also Fig. S2D). As expected, immunofluorescence staining showed high percentages of SOX2-positive (SOX2^+^) and Olig2^+^ tumor cells (stem cell markers) within tumor tissues from the mock infection group (Fig. 2E; see also Fig. S3A, bottom panels), whereas the proportions of SOX2^+^ and Olig2^+^ glioma cells were significantly lower in tumor tissues from the ZIKV-LAV-treated mice (Fig. 2E; see also Fig. S3A, top panels). The ZIKV E protein was clearly detected in tumor tissues from the ZIKV-LAV-administered mouse brain but was not frequently detected in normal brain tissue or glioma tissue from animals in the mock infection group (Fig. 2E; see also Fig. S3A).

10.1128/mBio.01683-18.3 FIG S3 ZIKV infection decreased GSC growth and induced cell death. Three-week-old BALB/c nude mice transplanted with 387 GSCs mixed with ZIKV-LAV or RPMI 1640 (mock) were sacrificed on day 23 postimplantation. The brain tissues were collected and cryosectioned. (A) Immunofluorescence staining of cryosections for the ZIKV E protein (green), Olig2 (red), and DAPI (blue). (B) The percentages of GSCs in tumor tissues are shown as means ± SD (*n* = 3). **, *P* < 0.01. (C) TUNEL staining of apoptotic cells in tumor tissues. (D) The percentages of apoptotic cells in tumor tissues are shown as means ± SD (*n* = 3). **, *P* < 0.01. Download FIG S3, TIF file, 2.3 MB. Copyright © 2018 Chen et al. 2018 Chen et al. This is an open-access article distributed under the terms of the Creative Commons Attribution 4.0 International license.

To examine whether the decrease in GSC populations was due to increased cell death, we performed cleaved caspase 3 (Cas3) staining and terminal deoxynucleotidyltransferase-mediated dUTP-biotin nick end labeling (TUNEL) assays on the brain tumor sections. More-extensive apoptosis was induced in the ZIKV-LAV-inoculated mice than in the animals receiving mock treatment (Fig. 2F; see also Fig. S3C). Altogether, these results suggest that ZIKV-LAV eliminates GSCs and their development into human GBM by inducing extensive apoptosis in an orthotopic mouse model.

### ZIKV-LAV has tumoricidal activity in GBM bulk cells.

**FIG 3:**
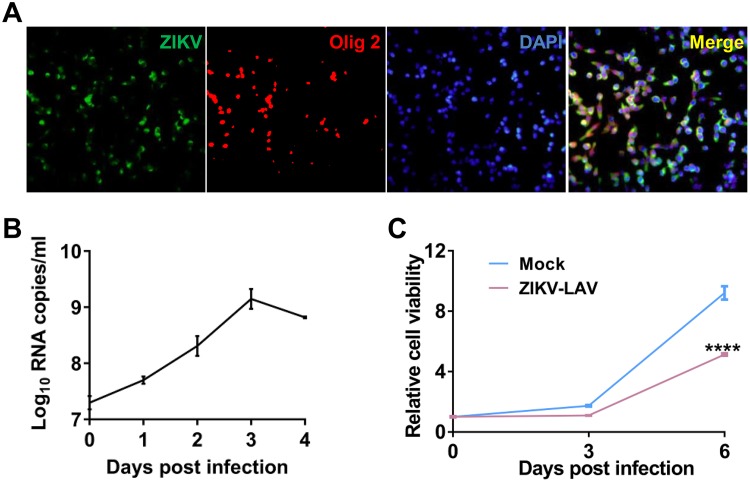
ZIKV-LAV has tumoricidal activity in glioblastoma bulk cells. (A) Immunofluorescence staining of glioblastoma bulk cells from 4121 GSC-implanted mice infected with ZIKV-LAV (MOI of 0.1). Bulk GBM cells were seeded in 24-well plates at a density of 1 × 10^5^ per well and infected with ZIKV-LAV at an MOI of 0.1. Immunofluorescence staining was performed on day 3 postinfection. ZIKV E protein (green), Olig2 (red), and DAPI (blue) are shown. (B) Growth curves of ZIKV-LAV in bulk cells (MOI of 0.1). Viral RNA copies in culture fluids were analyzed by RT-qPCR at the indicated time points. (C) Bulk cells were seeded in 96-well plates at a density of 2,000 per well and infected with ZIKV-LAV at an MOI of 10. Cell viability was detected by the Cell Titer-Glo assay at the indicated time points postinfection. Statistical analysis was performed by two-way ANOVA. ****, *P* < 0.0001.

To further determine the *ex vivo* tumoricidal activity of ZIKV-LAV, GBM bulk cells were isolated from 387 GSC-implanted mice and subjected to ZIKV-LAV infection. Immunostaining was performed on day 3 postinfection, and ZIKV preferentially infected the Olig2^+^ bulk tumor cells (Fig. 3A). Real-time quantitative PCR (RT-qPCR) analysis showed that ZIKV-LAV effectively replicated in bulk tumor cells, peaking on day 3 postinfection (Fig. 3B). Cell viability assays showed that ZIKV-LAV infection exerted direct oncolytic effects on GBM bulk cells (Fig. 3C).

### ZIKV-LAV preferentially infects and kills GSCs.

**FIG 4:**
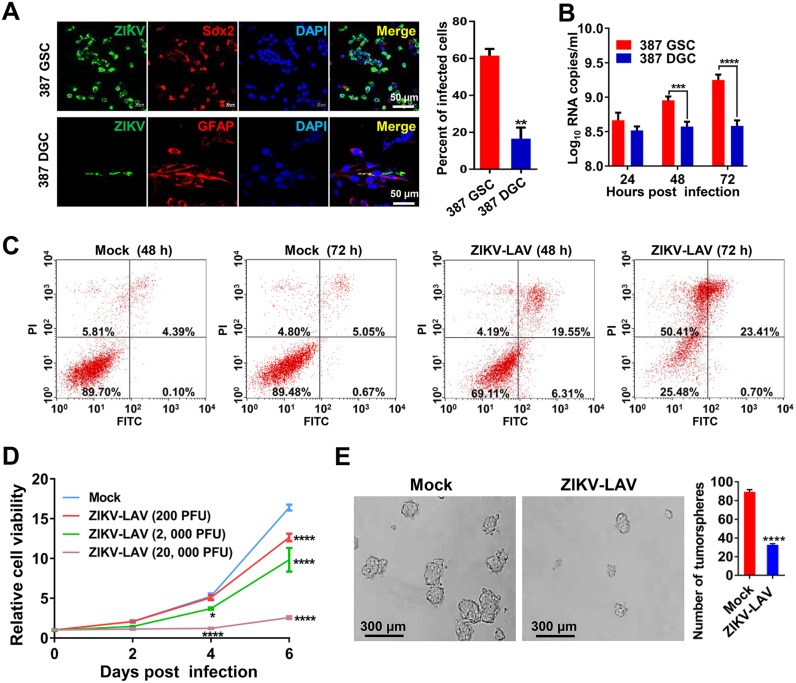
ZIKV-LAV preferentially infects and kills GSCs and impairs tumorsphere formation. (A) Immunofluorescence staining of ZIKV-LAV-infected 387 GSCs and DGCs for viral E protein (green), SOX2 or GFAP (red), and DAPI (blue) on day 3 postinfection (MOI = 0.1). (Right panel) Percentages of infected cells. Statistical analysis was performed using unpaired Student’s *t* tests. **, P < 0.01. (B) Growth curves of ZIKV-LAV in 4121 GSCs and DGCs (MOI of 0.1). Viral RNA copies in culture medium were analyzed by RT-qPCR at the indicated time points. Statistical analysis was performed by two-way ANOVA. ***, P < 0.001. ****, P < 0.0001. (C) Analysis of cell death induced by ZIKV-LAV infection. Briefly, 387 GSCs were infected with ZIKV-LAV (MOI of 1), collected at the indicated time points, and subjected to the cell death assay by flow cytometry. (D and E) GSCs were seeded in 96-well plates at a density of 2,000 per well, immediately infected with the indicated dose of ZIKV-LAV, and subjected to cell viability analysis using the Cell Titer-Glo assay at the indicated time points (D) and the tumorsphere assay on day 5 postinfection (E). Statistical analysis was performed by two-way ANOVA. *, P < 0.05; ****, P < 0.0001.

It was previously shown that wild-type ZIKV has a specific tropism for GSCs but not for differentiated glioma cells (DGCs) (16). To verify whether the vaccine virus retained this unique phenotype, we further characterized the *in vitro* oncolytic activity of ZIKV-LAV in both GSCs and DGCs. We found that approximately 60% of the SOX2^+^ 387 GSCs and 70% of the SOX2^+^ 4121 GSCs were infected by ZIKV-LAV. In contrast, only approximately 20% of the glial fibrillary acidic protein (GFAP)-positive cells (representing differentiated cells) were infected by ZIKV-LAV in their corresponding DGCs (Fig. 4A; see also Fig. S4A). ZIKV-LAV genomic RNA accumulated within 3 days postinfection in both 387 GSCs and 4121 GSCs, whereas no significant increase in viral RNA levels was observed in the infected DGCs (Fig. 4B; see also Fig. S4B). In addition, a flow cytometry assay showed that ZIKV-LAV infection induced extensive cell apoptosis (as revealed by staining with annexin V) in GSCs (Fig. 4C; see also Fig. S4C). Furthermore, standard cell viability and tumorsphere formation assays showed that ZIKV-LAV inhibited GSC growth in a dose-responsive manner (Fig. 4D; see also Fig. S4D) and suppressed GSC tumorsphere formation (Fig. 4E; see also Fig. S4E). These *in vitro* results, together with the *in vivo* and *ex vivo* results described above, demonstrate that ZIKV-LAV retains a tropism for GSCs and tumoricidal ability similar to that of wild-type ZIKV.

10.1128/mBio.01683-18.4 FIG S4 ZIKV-LAV preferentially infected GSCs, induced cell death, and abolished tumorsphere formation. (A) Immunofluorescence staining of ZIKV-LAV-infected 4121 GSCs and DGCs for viral E (green) and SOX2 or GFAP (red) and DAPI (blue) on day 3 postinfection (MOI of 0.1). (Right panel) Percentages of infected cells. Statistical analysis was performed using unpaired *t* tests. *, P  < 0.05. (B) Growth curves of ZIKV-LAV in 4121 GSCs and DGCs (MOI of 0.1). Viral RNA copies in the supernatant were analyzed by RT-qPCR at the indicated time points. Statistical analysis was performed by two-way ANOVA. **, P  < 0.01; ****, P  < 0.0001. (C) Analysis of GSC death induced by ZIKV-LAV infection. Type 4121 GSCs were infected with ZIKV-LAV at an MOI of 1. Cells were collected at the indicated time points and subjected to cell death assays by flow cytometry. (D and E) Cell viability and tumorsphere formation. GSCs were seeded in 96-well plates at a density of 2,000 per well and infected with the indicated dose of ZIKV-LAV. (D) The levels of viability of the infected 4121 GSCs were measured by the Cell Titer-Glo assay at the indicated time points. Statistical analysis was performed by two-way ANOVA. *, P  < 0.05; ***, *P*  < 0.001; ****, *P*  < 0.0001. (E) The tumorsphere values were calculated on day 5 postinfection. Statistical significance was analyzed by two-way ANOVA. ****, *P* < 0.0001. Download FIG S4, TIF file, 2.7 MB. Copyright © 2018 Chen et al. 2018 Chen et al. This is an open-access article distributed under the terms of the Creative Commons Attribution 4.0 International license.

### ZIKV infection triggers antiviral immunity and inflammation in GSCs.

**FIG 5:**
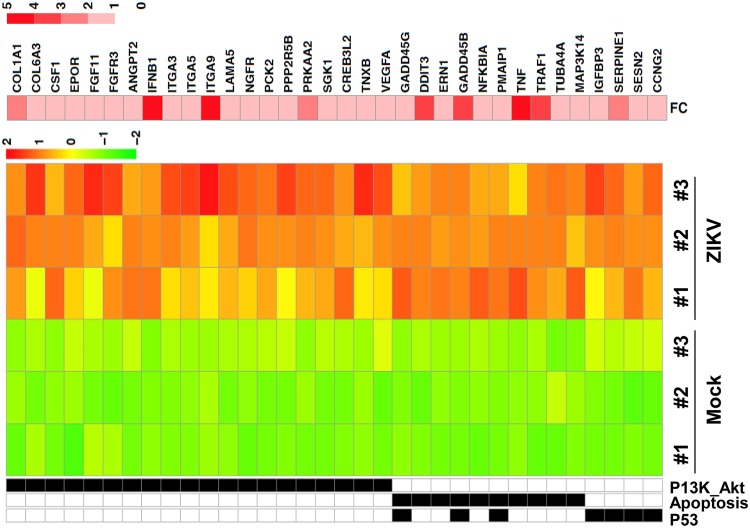
Relative expression of DEGs from the cell cycle, p53 signaling, apoptosis, and PI3K-Akt signaling pathways in ZIKV-infected GSCs. Type 4121 GSCs were infected with ZIKV (MOI of 1) for 48 h, and total RNAs were extracted and subjected to RNA-seq. Fold change (FC) values between the infected and mock-infected groups are presented in the log2 format. The other color keys are based on the Z-scores corresponding to the number of reads per kilobase per million mapped reads (RPKM).

**FIG 6:**
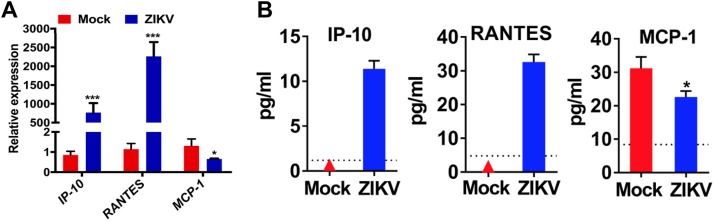
Specific cytokine responses in ZIKV-infected GSCs. Type 4121 GSCs were infected with ZIKV (MOI of 1) or PBS. Culture supernatants were harvested at 72 h postinfection. (A) Comparison of the mRNA levels of the IP-10, RANTES, and MCP-1 genes in the ZIKV-infected and mock-infected 4121 GSCs. (B) Protein levels of the cytokines IP-10, RANTES, and MCP-1 in 4121 GSCs infected with ZIKV or PBS (mock). The triangle symbols denote no detectable cytokines. The dotted lines denote the lowest detectable value of cytokines.

To reveal the underlying oncolytic mechanisms of ZIKV-LAV, we analyzed the global transcriptomic response in ZIKV-infected 4121 GSCs using transcriptome sequencing (RNA-seq). Differentially expressed genes (DEGs) and pathway analysis showed that ZIKV infection triggered strong antiviral immunity and inflammation responses, including the interferon (IFN), NF-κB, and tumor necrosis factor (TNF) signaling pathways (Fig. S5 and S6). These results are consistent with previous findings reported by Chen and coworkers (27). The relative gene expression levels of multiple DEGs from the cell cycle, p53, apoptosis, and phosphatidylinositol 3-kinase (PI3K)-Akt signaling pathways, which are related to cell differentiation, proliferation, or cell death, were significantly upregulated in ZIKV-infected 4121 GSCs (Fig. 5). We further validated the RNA-seq data by RT-qPCR. Selected genes blocking cell growth (DDIT3, GADD45B, and CDKN2B) were significantly upregulated by ZIKV infection (Fig. S6D). Interestingly, ZIKV infection dramatically upregulated gamma interferon (IFN-γ)-inducible protein 10 (IP-10) and RANTES and downregulated MCP-1 (Fig. 6A). In agreement with the result described above, the multiple-cytokine Luminex assay showed that ZIKV infection significantly enhanced the production of IP-10 and RANTES and reduced the production of MCP-1 in GSCs (Fig. 6B; see also Fig. S7). These results indicate that specific cytokine responses and interferon signaling collectively contribute to the oncolytic effects of ZIKV in GSCs.

10.1128/mBio.01683-18.5 FIG S5 ZIKV infection triggered antiviral immunity, inflammation, cell cycle arrest, and GSC apoptosis. Type 4121 GSCs were infected with ZIKV at an MOI of 1. Total RNA was extracted at 48 h postinfection. (A) Relative gene expression profiles of the following antiviral immunity pathways are presented: type I IFN signaling pathway, negative regulation of the viral genome replication pathway, and defense response to the virus pathway. (B) Relative gene expression profiles of the following inflammation pathways: the NF-κB and TNF signaling pathways. Download FIG S5, TIF file, 2.8 MB. Copyright © 2018 Chen et al. 2018 Chen et al. This is an open-access article distributed under the terms of the Creative Commons Attribution 4.0 International license.

10.1128/mBio.01683-18.6 FIG S6 Differentially expressed genes (DEGs) and pathway analysis in mock-infected and ZIKV-infected GSCs. (A) Gene expression profile of the top upregulated genes (*P* ≤ 0.05 and log2 FC ≥ 3). (B and C) GO biological process enrichment analysis (B) and KEGG pathway analysis (C) of all DEGs. (D) Selected genes related to cell growth or apoptosis in mock-infected and ZIKV-infected GSC cells were verified by qPCR. Download FIG S6, TIF file, 2.6 MB. Copyright © 2018 Chen et al. 2018 Chen et al. This is an open-access article distributed under the terms of the Creative Commons Attribution 4.0 International license.

10.1128/mBio.01683-18.7 FIG S7 ZIKV infection induced cytokine expression in GSCs. Type 4121 GSCs were infected with ZIKV (MOI of 1) or PBS (mock). The culture supernatants were harvested at 72 h postinfection. A heat map representing the levels of 25 cytokines expressed by the mock-infected or ZIKV-infected type 4121 GSCs is shown. In the heat map, all cytokines present at levels below the detectable level were assigned a value of 0. Download FIG S7, TIF file, 1.3 MB. Copyright © 2018 Chen et al. 2018 Chen et al. This is an open-access article distributed under the terms of the Creative Commons Attribution 4.0 International license.

## DISCUSSION

A recent study demonstrated that ZIKV had oncolytic activity against GBM in a mouse glioma model (16). However, applying this oncolytic virotherapy to clinical treatment requires two major improvements. First, wild-type ZIKV needs to be modified such that its neurovirulence is reduced to ensure its safety in treating glioma patients. Second, the modified safe ZIKV should retain its tropism and oncolytic activity against glioma cells. In this study, we demonstrated that a rationally engineered live attenuated ZIKV vaccine candidate in which a 10-nucleotide region is deleted from the 3′UTR of the viral genome was safe when intracerebrally injected into the mouse brain, as indicated by the absence of disease, histopathology, or behavioral abnormalities. Importantly, treatment with ZIKV-LAV potently killed human GSCs *in vitro*, eliminated the development of GSCs into glioma in an orthotopic xenograft mouse model, and prolonged the survival of animals. It should be noted that, compared with a recent study (16), the current study used (i) a well-developed ZIKV vaccine candidate that is currently transitioning into clinical development (7) and (ii) a human GSC orthotopic xenograft mouse model to demonstrate its efficacy.

A common strategy to ensure the safety of oncolytic viruses is to adopt vaccine strains by taking advantage of their attenuated nature. On the basis of this idea, three oncolytic virus platforms, i.e., measles virus vaccine strain Edmonston B, poliovirus vaccine strain Sabin, and several vaccinia virus vaccine strains, have entered clinical trials (14). Recombinant poliovirus containing the internal ribosomal entry site (IRES) from rhinovirus was recently shown to improve the rate of survival of grade IV malignant glioma patients (15). For our ZIKV-LAV, we previously showed that treatment of 1-day-old CD-1 mice with the vaccine candidate by intracranial injection was safe and that the virus was unable to infect Aedes aegypti mosquitoes because of its decreased level of viral RNA synthesis and increased sensitivity to type I interferon inhibition (17). Strikingly, testing our ZIKV-LAV in the 1-day-old mice showed that it was safer than the YFV 17D and JEV SA14-14-2 vaccines (two licensed live attenuated flavivirus vaccines). In this study, we further evaluated its safety from other aspects. Like the test in CD-1 mice, intracranial inoculation of 3-week-old BALB/c nude mice with 10,000 PFU of ZIKV-LAV caused no mortality and even no weight loss, whereas infection with the same dose of wild-type ZIKV was lethal. Although limited viral replication was observed in brains inoculated with ZIKV-LAV, no pathological damage was observed in the mouse brains, and assays showed that the virus did not invade other organs or cause viremia at days 6 to 18 posttreatment. These results suggest that the attenuated virus may be incapable of shedding from the inoculated animals into the environment or infecting bystanders (e.g., via sexual transmission), providing a safety feature for treated individuals and the public. However, we could not exclude the possibility that early viremia may have occurred but had been cleared by day 6 posttreatment. Furthermore, the performances of C57 mice intracranially inoculated with ZIKV-LAV or phosphate-buffered saline (PBS) during behavioral evaluations, including the open field test, tail suspension test, elevated plus maze test, and rota-rod test, were not different. Taken together, our results demonstrate that ZIKV-LAV has an excellent safety profile and is thus a potential virotherapeutic agent for GBM patients.

An oncolytic virus must display a fine balance between efficacy and safety to be successful. To determine whether the virus retains oncolytic activity after being attenuated, we mixed patient-derived GSCs with ZIKV-LAV or RPMI 1640 and then transplanted the mixture into the brains of BALB/c nude mice. Note that because patient-derived GSCs maintain patient-specific genetic and phenotypic alterations, the evaluation of oncolytic efficiency with GBM models constructed using patient-derived GSCs in the current study represents a step closer to the treatment of human GBM than the mouse glioma models reported by Zhu and colleagues (16). The results of transplantation of the premixture of ZIKV-LAV and GSCs resemble the prevention of GBM recurrence in the clinical setting, during which the main part of the tumor is removed by surgical resection first and then temozolomide chemotherapy and radiation are applied to eliminate residual tumor cells (28, 29). Using this model, we found that the ZIKV-LAV-treated mice survived longer than the untreated mice, demonstrating that ZIKV-LAV retains its oncolytic activity against patient-derived GSCs *in vivo*.

To reveal the mechanism underlying the observed oncolytic activity, we analyzed the transcription profiles of mock-infected and ZIKV-infected GSCs. DEG assays and pathway analysis showed that ZIKV infection triggered strong activation of the TNF signaling pathway (see Fig. S5 in the supplemental material) and upregulated the expression of several cytokines, including IP-10 (CXCL10). These results were further corroborated by the cytokine Luminex assay data. IP-10/CXCL10 can recruit CXCR3^+^ T cells, including CD8^+^ T cells, which are important for the control of tumor growth, via the CXCR3 receptor expressed on activated T cells (30). Nishimura et al. also reported that IP-10 plays a critical role in the homing of Tc1 into the central nervous system to inhibit tumors (31), and IP-10 has been shown to inhibit tumoral angiogenesis (32). Thus, ZIKV-LAV treatment may lead not only to direct cell death but also to the activation of antitumor immunity *in vivo*. Because our current model was built in BALB/c nude mice, which are T cell deficient, the oncolytic efficiency contributed by T cell immunity could not be revealed. Therefore, a patient-derived GSC glioma model in more highly immunocompetent mice is needed to better reveal the oncolytic efficiency of ZIKV-LAV. However, one limitation of the mouse model used here is that because the GSCs used are of human origin, ZIKV-LAV may spread differently to normal mouse tissue (i.e., mouse brain) than to human tissue (i.e., spread from human tumor to human normal brain tissue) during tumor treatment.

In summary, the current report represents major progress toward the development of ZIKV as a novel virotherapeutic candidate for the treatment of human GBM. We demonstrated that a live attenuated ZIKV vaccine candidate has the excellent safety profile required for cerebral virotherapy and retains potent oncolytic efficacy in glioma models built with patient-derived GSCs.

## MATERIALS AND METHODS

### Cell lines.

GSCs (specimens 387 and 4121) were originally isolated and characterized from human GBM surgical specimens as previously described (26) and cultured as neurospheres in neurobasal complete media (Gibco) supplemented with 1× B27 (Gibco) (without vitamin A), 2 mM l-glutamine (MacGene), 1 mM sodium pyruvate (MacGene), 10 ng/ml basic fibroblast growth factor (R&D Systems), and 10 ng/ml epidermal growth factor (R&D Systems). DGCs were differentiated from GSCs via the withdrawal of epidermal growth factor and fibroblast growth factor and addition of 10% fetal bovine serum (FBS; MacGene). DGCs were cultured in Dulbecco’s modified Eagle’s medium (MacGene) containing 10% FBS. Expression of the GSC marker SOX2/Olig2 and the differentiation marker GFAP was characterized using Western blotting or immunofluorescence staining. All cells were incubated at 37°C in a humidified incubator supplemented with 5% CO_2_.

### Viruses.

The wild-type ZIKV strain used in this study was originally isolated by our laboratory from a Chinese patient returning from Venezuela in 2016 (33). The live attenuated vaccine strain of ZIKV (ZIKV-LAV) was generated by reverse genetic technology, and the characterization of ZIKV-LAV was described previously (17). The JEV-LAV used in this study was obtained from Chengdu Institute of Biological Products (China).

### Mouse experiments.

The mouse strains used in this study included BALB/c nude mice (3 weeks old, female) and C57 mice (4 weeks old, female); both mouse strains were purchased from Beijing Vital River Laboratory Animal Technology Co., Ltd. All animal experiments were approved by the Animal Experiment Committee of Laboratory Animal Center, AMMS, China (IACUC-13-2016-001).

### Safety evaluation of ZIKV-LAV in mice.

For the survival and weight change study, groups of BALB/c nude mice were intracerebrally inoculated with 10,000 PFU of ZIKV-LAV, JEV-LAV, or ZIKV. PBS injection was used as a control. Mice were weighed and monitored daily to assess body weight changes and mortality.

To study the tissue distribution of ZIKV-LAV and ZIKV, sera and major tissues (including brains, livers, spleens, kidneys, hearts, and lungs) of mice intracerebrally inoculated with ZIKV-LAV (10,000 PFU/mouse) or ZIKV (10,000 PFU/mouse) were harvested on days 6, 12, and 18 after infection (*n* = 3 per time point) for the detection of viral RNA by RT-qPCR. The primers and probe used in this study were described previously (34).

For histological examination, the brains of BALB/c nude mice intracerebrally inoculated with 10,000 PFU of ZIKV-LAV or PBS were harvested on day 7 after inoculation and then prepared into 8-μm-thick paraffin sections. The paraffin sections were stained with hematoxylin and eosin.

### Behavioral evaluations of mice infected with ZIKV-LAV.

Groups of C57 mice were inoculated with ZIKV-LAV (10,000 PFU per mouse) or PBS via an intracerebral route. On day 14 postinoculation, the open field, tail suspension, rota-rod, and elevated plus maze tests were performed accordingly.

For the open field test, each mouse was placed in the center of a darkened white box (30 by 30 by 40 cm) and monitored using an infrared video tracking system (Ethovision XT 9.0; Noldus Information Technology) for 5 min. A 15-by-15-cm square in the center of the box was defined as the “zone,” and the peripheral arena was defined as the “residual.” The distance traveled and time spent in the zone and residual were recorded for further analysis.

For the tail suspension test, animals were suspended 50 cm above the floor by hanging approximately 1 cm from the tip of their tail. The time that the mice remained immobile was quantified during a test period of 6 min. Mice were considered immobile only when they hung passively and stayed completely motionless. A camera was mounted facing the tail suspension test arena, and all the test events were recorded. Two experienced observers independently scored the behavior, and an average of the two scores was used as the final score.

For the elevated plus maze test, the structure used was a “plus”-shaped maze elevated 60 cm above the floor that consists of two closed arms surrounded by nontransparent walls (20 cm in height) and two open arms (5 by 35 cm). Each mouse was placed in the center (5 by 5 cm) of the maze, facing one of the closed arms. During the 5-min test period, the movement of the mouse was recorded by video capture software (Anymaze). The total number of entrances into the open arms and the amount of time spent in the open arms were quantified.

For the rota-rod test, each mouse was placed on a rotating spindle that accelerated from 4 rpm to 40 rpm within 5 min and remained at 40 rpm for one additional minute. All animals were subjected to one session per day for 3 days, and the amount of time spent on the rod was recorded for quantitative analysis.

### Construction of the GBM orthotopic xenograft model using patient-derived GSCs.

A total of 50,000 luciferase-labeled GSCs (type 387 or 4121) were co-implanted with 10,000 PFU of ZIKV-LAV or RPMI 1640 intracerebrally into the right frontal lobe of a nude mouse. After implantation, bioluminescence imaging was performed using a charge-coupled-device camera (Xenogen Corp., Alameda, CA, USA) at the indicated time points as described previously (35). Briefly, 1.5 mg of the substrate d-Luciferin sodium salt (Gold Biotechnology) was administered intraperitoneally to each mouse, and images were acquired 9 min later for 60 s. To quantify the amount of light emitted from the tumor, regions of interest (ROIs) were manually defined after imaging, and the photon flux was calculated (in photons/second/square centimeter/steradian) using Living Image 3.0 (Caliper Life Sciences, Alameda, CA, USA). For experiments assessing survival, mice were monitored until the last mouse showed neurological signs. When any mouse showed neurological signs, selected mouse brains were harvested and prepared in 5-μm-thick cryosections. The cryosections were stained with hematoxylin and eosin and subjected to histological examination.

### Immunofluorescence staining of brain tissues.

Brain cryosections were incubated with a primary mouse anti-ZIKV E protein antibody (Abcam) (1:500 dilution), goat anti-SOX2 antibody (R&D) (1:500 dilution), or goat anti-Olig2 antibody (R&D) (1:50 dilution) overnight at 4°C. The sections were then incubated with Alexa Fluor 488-conjugated anti-mouse and 594-conjugated anti-goat secondary antibodies (Thermo Fisher) (1:300 dilution) for 2 h at 37°C. Finally, the sections were incubated with DAPI (4′,6-diamidino-2-phenylindole) (1:1,000 dilution) for 10 min. Images were acquired by confocal laser scanning microscopy (Zeiss).

### Assessment of apoptosis in tumor tissues.

Apoptosis in tumor tissue was detected by cleaved caspase 3 staining and a DeadEnd colorimetric TUNEL system kit (Promega). For cleaved caspase 3 staining, the brain cryosections were incubated with the primary mouse anti-ZIKV E protein antibody or rabbit anti-cleaved caspase 3 antibody (CST) (1:200 dilution) and Alexa Fluor 488-conjugated anti-mouse and 594-conjugated anti-rabbit secondary antibodies (Thermo Fisher) (1:300 dilution). Finally, the sections were incubated with DAPI (1:1,000 dilution) for 10 min. A DeadEnd colorimetric TUNEL system kit (Promega) was utilized according to the manufacturer’s instructions. Images were acquired by confocal laser scanning microscopy (Zeiss).

### Bulk cell isolation.

A total of 1 × 10^6^ 4121 GSCs were subcutaneously implanted into the flanks of BALB/c nude mice. The xenografts were harvested 3 weeks after implantation, and the inner contents were centrifuged for 3 min at a speed of 1,000 × *g*. Cells obtained by centrifugation were treated with Accutase (Sigma) for 3 min. After being washed with PBS, bulk cells were cultured in Neurobasal complete media and subjected to the following assays.

### Viral titration and immunofluorescence assay (IFA).

Slides were placed in 24-well plates and then covered with Matrigel (BD). Bulk cells, GSCs, or DGCs were seeded at a density of 1 × 10^5^ cells per well. After 24 h, the cells were infected with ZIKV-LAV at a multiplicity of infection (MOI) of 0.1 for 1 h, and the culture medium was then refreshed. At the indicated times, the supernatants were collected for virus titration by RT-qPCR as described above. The bulk cells and GSCs were incubated with the same primary and secondary antibodies used for the immunofluorescence analysis of the brain cryosections. The DGCs were incubated with the mouse anti-ZIKV E protein (Abcam) (1:500 dilution) and rabbit anti-GFAP (Dako) (1:500 dilution) primary antibodies overnight at 4°C and then with Alexa Fluor 488-conjugated anti-mouse and 594-conjugated anti-rabbit secondary antibodies (Thermo Fisher) (1:300 dilution) for 2 h at 37°C. The cells were subsequently incubated with DAPI (1:1,000 dilution) for 10 min. Images were acquired by confocal laser scanning microscopy (Zeiss).

### Cell death analysis by flow cytometry.

GSCs were infected with ZIKV-LAV at an MOI of 1 and collected at 48 and 72 h postinfection. Flow cytometry was performed with an annexin V-fluorescein isothiocyanate (annexin V-FITC) apoptosis detection kit (Trevigen) according to the manufacturer’s instructions. In brief, cells were washed in Neurobasal medium, and 1 × 10^5^ cells were subsequently resuspended in 100 μl of binding buffer and incubated with 5 μl of propidium iodide (PI) and 5 μl of annexin V-FITC for 15 min in the dark at room temperature. Flow cytometric analysis was immediately performed using a flow cytometer (BD).

### Cell viability and tumorsphere formation assay.

Cells were seeded in 96-well plates at a density of 2,000 per well and subsequently infected with ZIKV-LAV at a dose of 200, 2,000, or 20,000 PFU per well. Cell viability was evaluated on the indicated days after infection with a Cell Titer-Glo luminescent cell viability assay kit (Promega) according to the manufacturer’s instructions. The tumorspheres were counted 5 days after infection.

### RNA-seq data acquisition, quality control, and processing.

GSCs were infected with ZIKV at an MOI of 1, and total cellular RNA was extracted at 48 h postinfection with a PureLink RNA minikit (Life Technologies). The RNA concentration was quantified using a Qubit 2.0 Fluorometer (Thermo Fisher), and the quality of the extracted RNA was evaluated using an Agilent Technology 2100 Bioanalyzer. RNA libraries were constructed using a TruSeq Stranded mRNA Sample Prep kit (Illumina) according to the manufacturer’s guidelines. The quantities and qualities of the libraries were also assessed by the use of the Qubit Fluorometer and Agilent 2100 Bioanalyzer, respectively, and their molar concentrations were validated by qPCR for library pooling. Libraries were sequenced on a HiSeq X10 platform using the paired-end 2 × 150-bp, dual-index format. For RNA-seq data analysis, first, trimmomatic was used to remove Illumina sequencing adapters within the raw reads of every sample, trim the low-quality bases of both read ends (with parameters LEADING:3 TRAILING:3 SLIDINGWINDOW:4:15), and remove reads less than 36 bp in length. Second, the clean reads were mapped to the human hg38 reference genome with STAR, and the alignment bam files were used as an HTSeq-count (command of the python package HTSeq) input to obtain the gene read counts. Finally, DESeq2 was used to identify DEGs (*P  ≤ *0.05, fold change [FC] ≥ 2) based on raw read counts. For DEGs, KEGG pathway and gene ontology (GO) biological process analyses were performed using Fisher’s exact test, and the enrichment *P* values were corrected by the Bonferroni method.

### RNA isolation, reverse transcription, and real-time qPCR.

GSCs were infected with ZIKV at an MOI of 1 in biological triplicate, and total cellular RNA was extracted at 48 h postinfection with a PureLink RNA minikit (Life Technologies). cDNA was generated from 1 mg of RNA using an IScript reverse transcription kit (Bio-Rad) according to the manufacturer’s instructions with random primers. qPCR analysis was performed using an iCycler thermocycler (Bio Rad). qPCR was performed using a Sensimix SYBR & Fluorescein kit (Bioline) according to the manufacturer’s instructions. Expression values were normalized to the RPL32 control, and fold induction was normalized to an untreated control using the threshold cycle (2^−ΔΔ^*^CT^*) method. The following gene primer sequences were used: for DDIT3, 5′-GGAAACAGAGTGGTCATTCCC-3′ (F) and 5′-CTGCTTGAGCCGTTCATTCTC-3′ (R); for GADD45B, 5′-TACGAGTCGGCCAAGTTGATG-3′ (F) and 5′-GGATGAGCGTGAAGTGGATTT-3′ (R); for CDKN2B, 5′-CGGGGACTAGTGGAGAAGGT-3′ (F) and 5′-CCCATCATCATGACCTGGAT-3′ (R); for IP-10, 5′-GTGGCATTCAAGGAGTACCTC-3′ (F) and 5′-TGATGGCCTTCGATTCTGGATT-3′ (R); for RANTES, 5′-CCAGCAGTCGTCTTTGTCAC-3′ (F) and 5′-CTCTGGGTTGGCACACACTT-3′ (R); for MCP-1, 5′-CAGCCAGATGCAATCAATGCC-3′ (F) and 5′-TGGAATCCTGAACCCACTTCT-3′ (R); and for RPL32, 5′-GAAACCCAGAGGGATTGACA-3′ (F) and 5′-GACGTTGTGGACCAGGAACT-3′ (R).

### Cytokine analysis.

The supernatants from ZIKV-infected GSCs were collected at 72 h postinfection for cytokine analysis with a Human Cytokine Magnetic 25-Plex panel (Life Technologies) according to the manufacturer’s instructions. The data were collected on a Luminex 200 instrument and analyzed by Luminex xPONENT software (Thermo Fisher).

### Statistical analysis.

All data are shown as means ± standard deviations (SD). Survival curves were analyzed by a log rank (Mantel-Cox) test. Data obtained from two groups at a single time point were analyzed by unpaired *t* tests. Measurements at multiple time points were analyzed by two-way analysis of variance (ANOVA). All statistical analyses were performed with GraphPad Prism 7.01 software.

### Data availability.

We declare that all relevant data are available from the corresponding author upon request. All sequence data generated by RNA-seq have been deposited into the Gene Expression Omnibus database (accession no. GSE114907).
