# Erratum for Chen et al., “Treatment of Human Glioblastoma with a Live Attenuated Zika Virus Vaccine Candidate”

**DOI:** 10.1128/mBio.00433-19

**Published:** 2019-04-02

**Authors:** Qi Chen, Jin Wu, Qing Ye, Feng Ma, Qian Zhu, Yan Wu, Chao Shan, Xuping Xie, Dapei Li, Xiaoyan Zhan, Chunfeng Li, Xiao-Feng Li, Xiaoling Qin, Tongyan Zhao, Haitao Wu, Pei-Yong Shi, Jianghong Man, Cheng-Feng Qin

**Affiliations:** a State Key Laboratory of Pathogen and Biosecurity, Institute of Microbiology and Epidemiology, Academy of Military Medical Sciences, Beijing, China; b State Key Laboratory of Proteomics, National Center of Biomedical Analysis, Beijing, China; c Guangzhou No. 8 People's Hospital, Guangzhou Medical University, Guangzhou, China; d Center for Systems Medicine, Institute of Basic Medical Sciences, Chinese Academy of Medical Sciences and Peking Union Medical College, Beijing, China; e Suzhou Institute of Systems Medicine, Suzhou, China; f Department of Neurobiology, Beijing Institute of Basic Medical Sciences, Beijing, China; g Department of Biochemistry and Molecular Biology, Department of Pharmacology and Toxicology, Sealy Center for Structural Biology and Molecular Biophysics, University of Texas Medical Branch, Galveston, Texas, USA; h Tianjin Key Laboratory of Risk Assessment and Control Technology for Environment and Food Safety, Institute of Environmental Medicine, Academy of Military Medical Sciences, Tianjin, China

## ERRATUM

Volume 9, issue 5, e01683-18, 2018, https://doi.org/10.1128/mBio.01683-18. The fourteenth author’s name was misspelled. The byline should appear as shown above.

