# Supplementary figures and images for: Treatment of Human Glioblastoma with a Live Attenuated Zika Virus Vaccine Candidate
Source: mBio. 2018 Sep 18;9(5):e01683-18. doi: 10.1128/mBio.01683-18 (PMC6143740; doi:10.1128/mBio.01683-18)

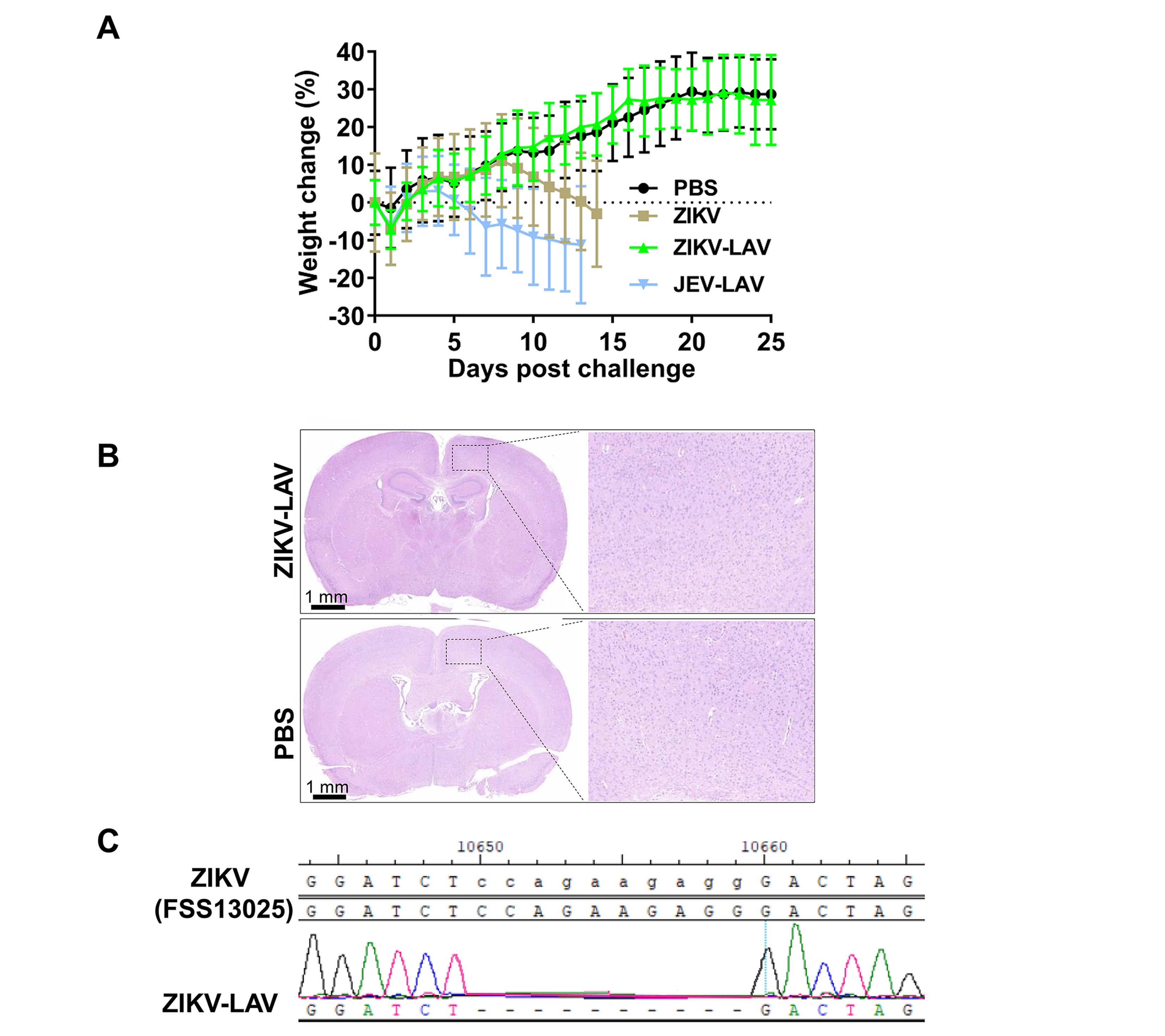

Supplement: FIG S1 [file mbo005184083sf1.tif]

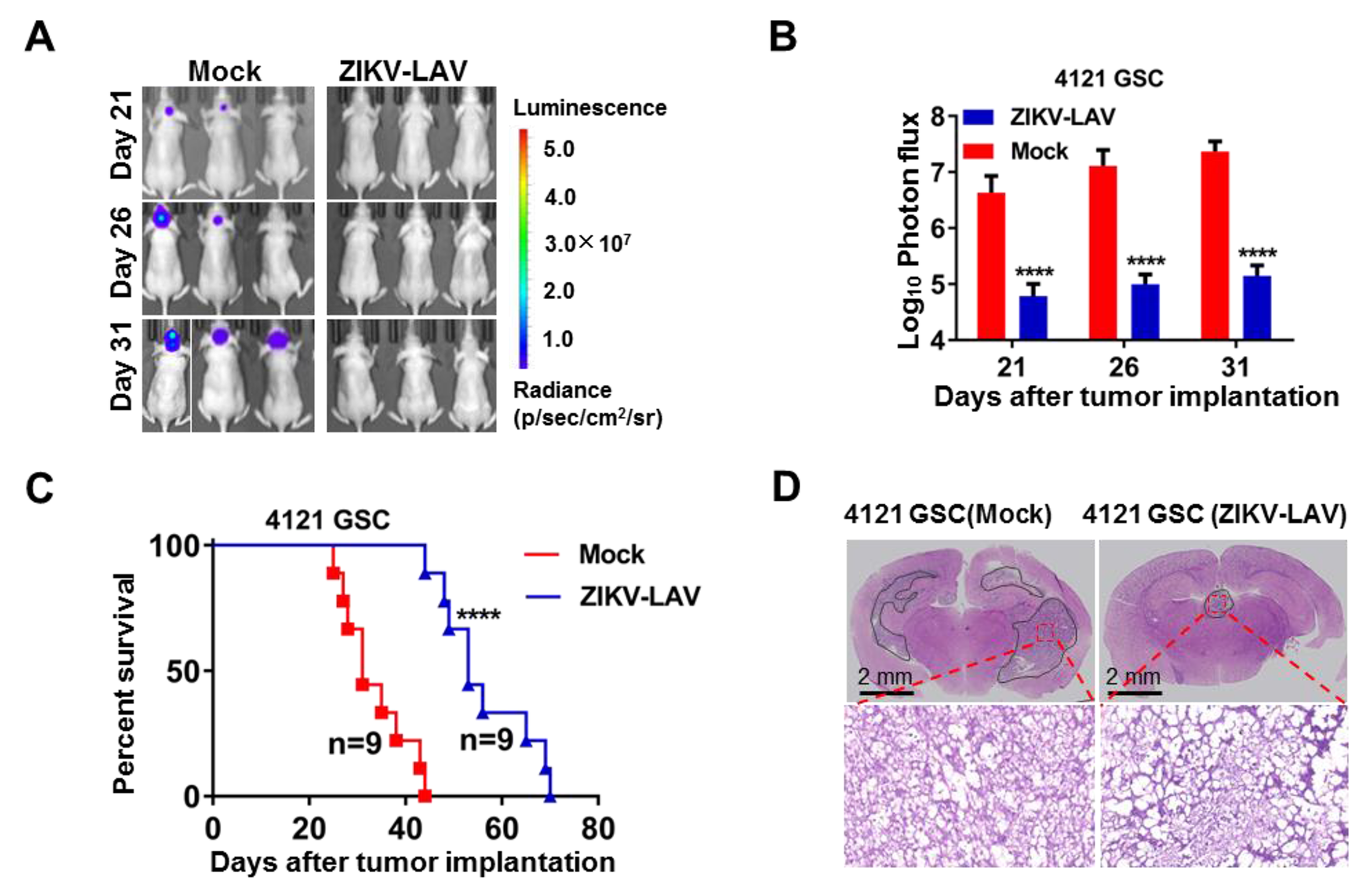

Supplement: FIG S2 [file mbo005184083sf2.tif]

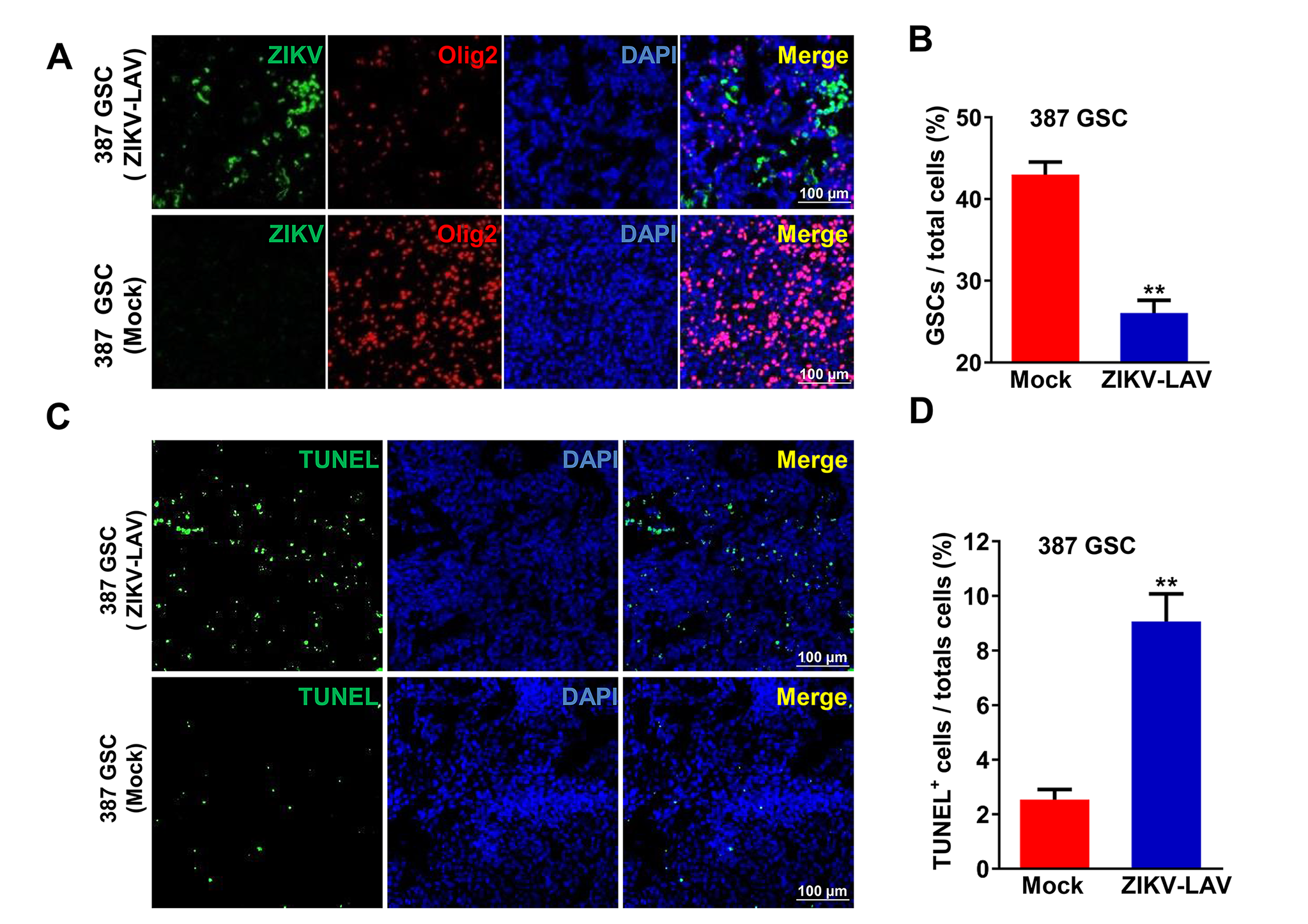

Supplement: FIG S3 [file mbo005184083sf3.tif]

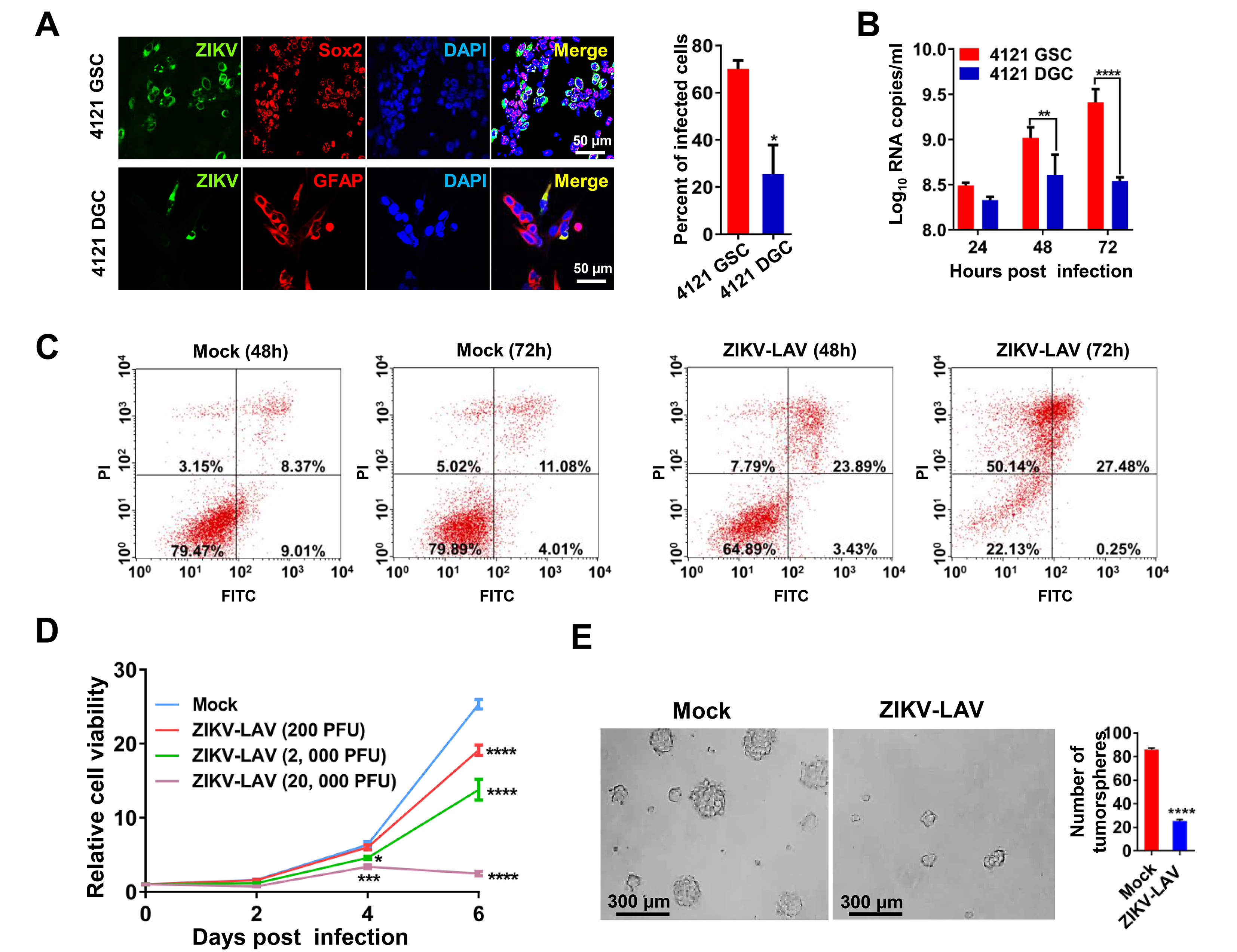

Supplement: FIG S4 [file mbo005184083sf4.tif]

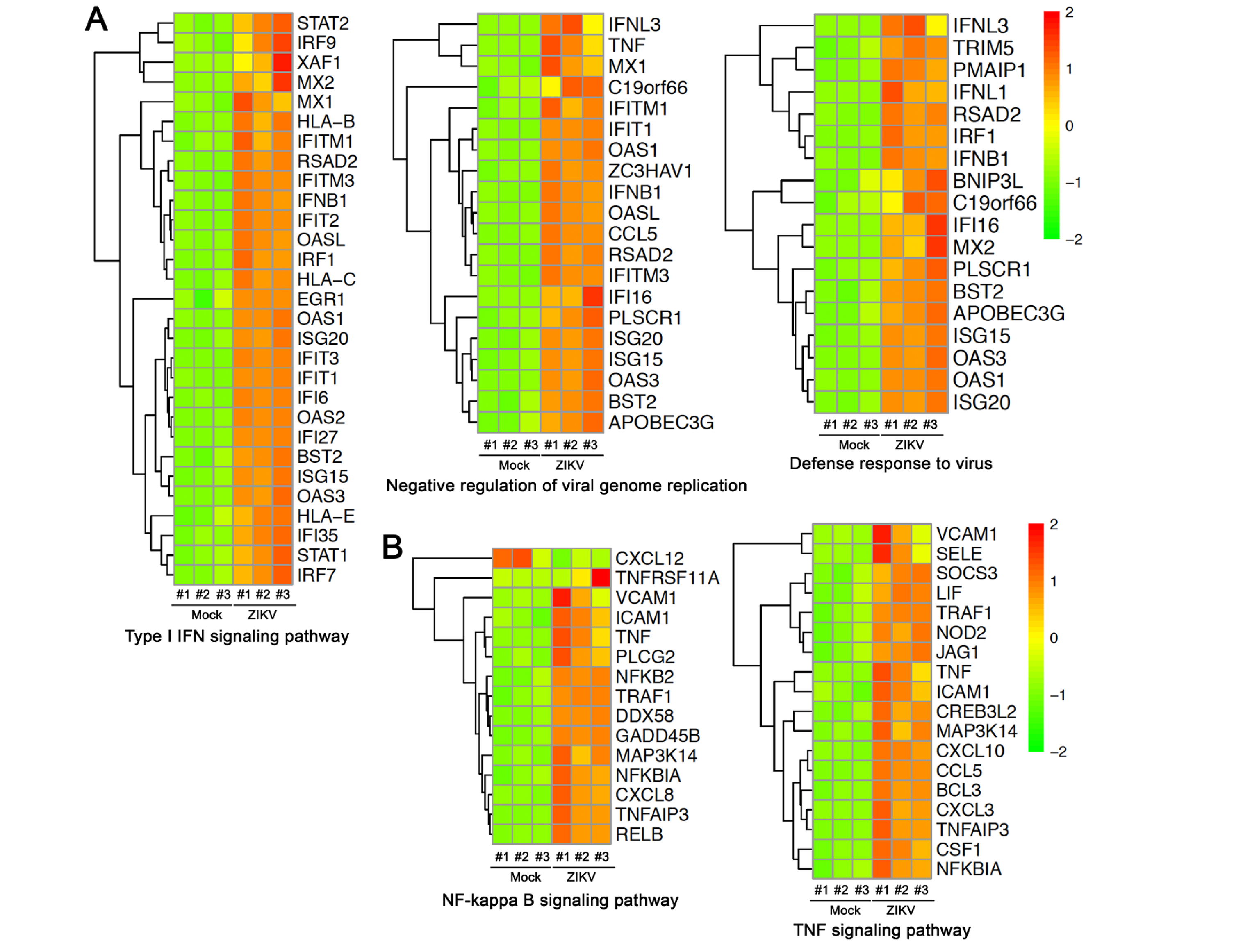

Supplement: FIG S5 [file mbo005184083sf5.tif]

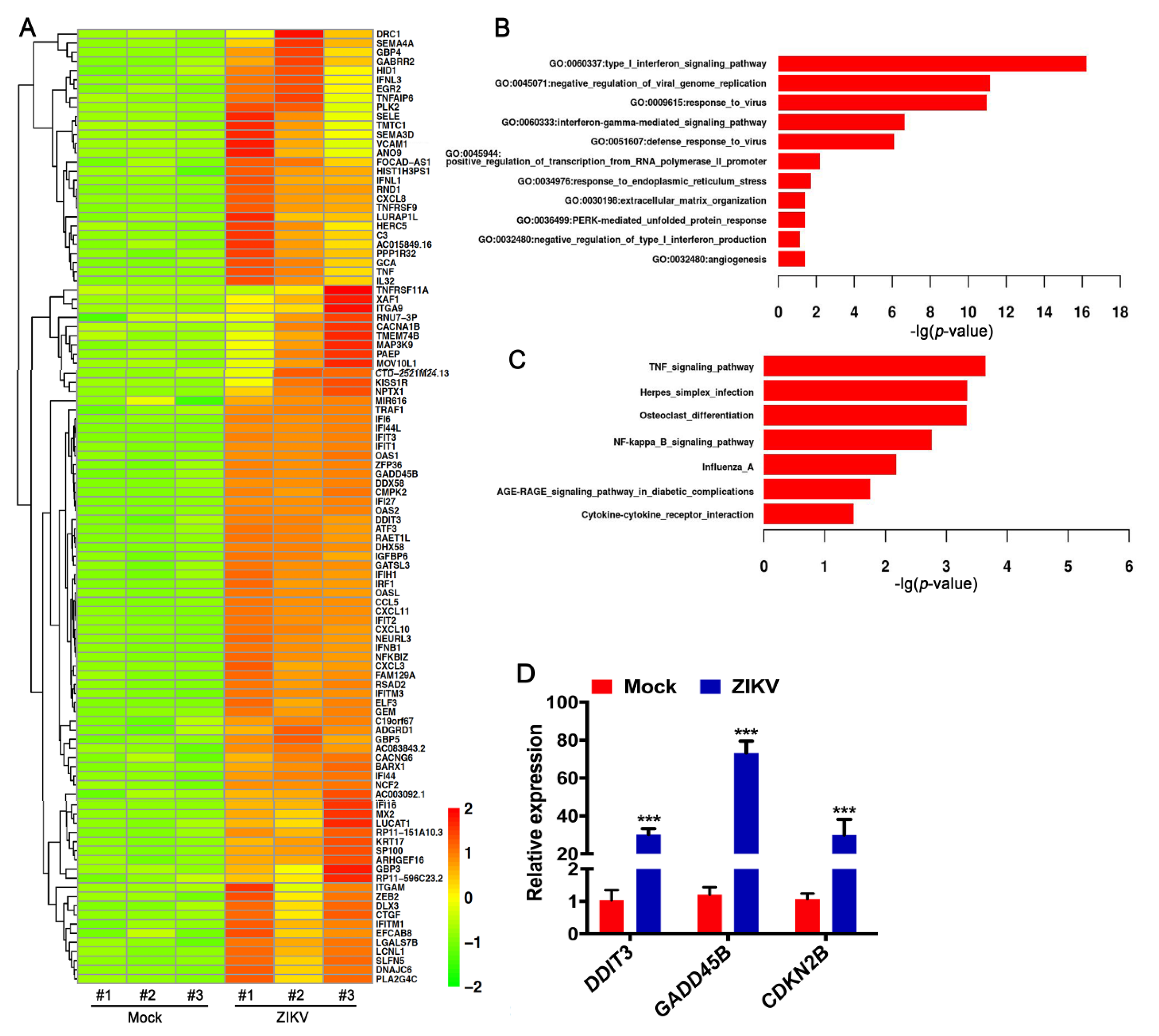

Supplement: FIG S6 [file mbo005184083sf6.tif]

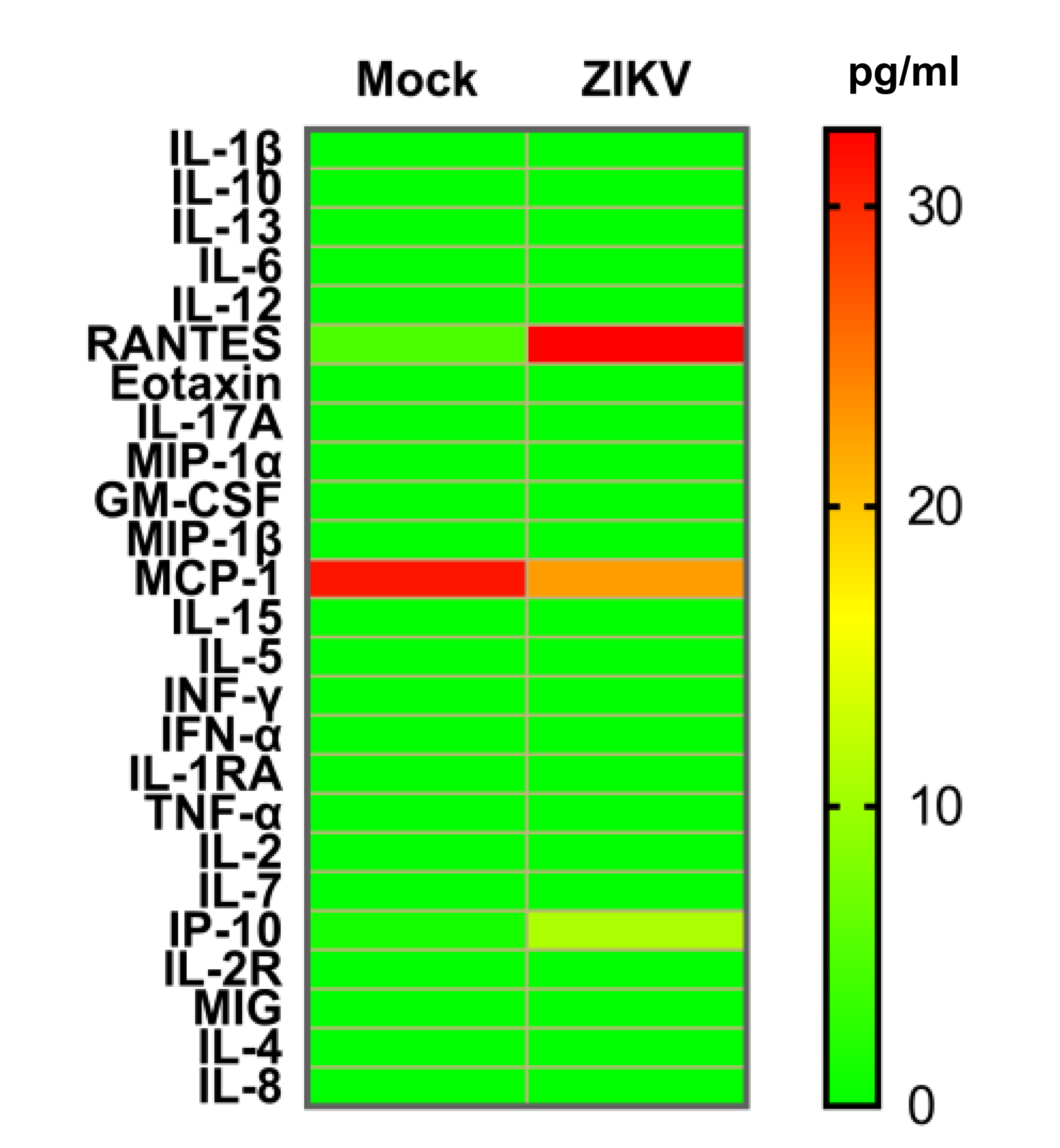

Supplement: FIG S7 [file mbo005184083sf7.tif]
